# PHYSICAL ACTIVITY MEDIATES LATIN DANCE PARTICIPATION AND FITNESS OUTCOMES IN OLDER LATINOS

**DOI:** 10.1093/geroni/igac059.2388

**Published:** 2022-12-20

**Authors:** Navin Kaushal, Guilherme Balbim, Michelle Jaldin, David Marquez

**Affiliations:** Indiana University, Indianapolis, Indiana, United States; University of British Columbia, Vancouver, British Columbia, Canada; University of Illinois Chicago, Chicago, Illinois, United States; University of Illinois Chicago, Chicago, Illinois, United States

## Abstract

**Background:**

The benefits of engaging in physical activity (PA) for older adults (OA) are well documented; however, participation rates remain low, especially among OA Latinos. Latin dance expresses and promotes culture among Latinos, and can be an effective approach to promote PA. However, the physical function and cardiorespiratory fitness (CRF) benefits of OA engaging in Latin dance have not been investigated. The purpose of this study was to test if PA from an 8-month dance trial yielded and explained improvements in physical function and CRF.

**Methods:**

The study analyzed physical function and CRF outcomes from the BAILA trial. Participants (n= 333) were Latinos (age 55+) who were randomized to a dance or control condition for an 8-month study. PA was assessed using the Community Healthy Activities Model Program for Seniors (CHAMPS), physical function was assessed with the short physical performance battery protocol (SPPB) and estimated CRF was assessed using the Jurca non–exercise test model. Results. ANCOVA models found significant change in SPPB total scores(F1, 331= 4.01, p=0.046) and estimated CRF (F1, 331= 7.66, p= 0.006) over eight months in favor of the dance group. Follow-up mediation models found MVPA to mediate between group and SBBP scores, (β= 0.05, 95% CI [0.0128, 0.1147]). MVPA also mediated between group and CRF, (β= 0.06, 95% CI [0.0164, 0.1197]). Conclusion. The study supports organized Latin dance programs to be effective for improving physical and cardiorespiratory benefits among older adults. The findings also encourage future investigations to promote PA in culturally relevant forms.

